# Foot posture as a biomechanical outcome measure following total knee arthroplasty in individuals with severe knee osteoarthritis

**DOI:** 10.1038/s41598-025-31351-0

**Published:** 2025-12-05

**Authors:** Saidan Shetty, Mohandas Rao KG, Sandeep Vijayan, G. Arun Maiya, Bincy M. George

**Affiliations:** 1 Department of Anatomy, Melaka Manipal Medical College - Manipal Campus, Manipal Academy of Higher Education (MAHE), Manipal, 576104 Karnataka India; 2 Division of Anatomy, Department of Basic Medical Sciences, Manipal Academy of Higher Education (MAHE), Manipal, 576104 Karnataka India; 3 Department of Orthopaedics, Kasturba Medical College (KMC) Manipal, Manipal Academy of Higher Education (MAHE), Manipal, 576104 Karnataka India; 4 Department of Physiotherapy, Centre for Podiatry & Diabetic Foot Care and Research, Manipal College of Health Professions (MCHP), Manipal Academy of Higher Education (MAHE), Manipal, 576104 Karnataka India

**Keywords:** Foot posture, Total knee arthroplasty, Total knee replacement, Knee osteoarthritis, Diseases, Health care, Medical research, Rheumatology

## Abstract

Total knee arthroplasty (TKA) is commonly performed to relieve pain and improve knee function in individuals with severe knee osteoarthritis (OA). Abnormal foot mechanics can influence overall biomechanics and affect post-surgical outcomes. This study aimed to evaluate foot posture as a biomechanical outcome before and after TKA and to explore its relationship with pain, functional performance, and quadriceps (Q) angle. Seventy-seven individuals with knee OA who underwent TKA, and 77 healthy controls were assessed. Foot posture was measured using the Foot Posture Index (FPI), pain using the Numerical Pain Rating Scale (NPRS), and function using the Lower Extremity Functional Scale (LEFS), Timed Up and Go (TUG), and 6-Minute Walk Test (6MWT). Q-angle was assessed as a structural alignment measure. Evaluations were conducted preoperatively and at 6 weeks, 3, 6, and 12 months postoperatively. Preoperative FPI was significantly higher in the OA group than controls (1.52 ± 2.5 vs. 0.70 ± 2.2; *p* = 0.03). At 6 months post-TKA, FPI remained higher than controls (*p* = 0.03), but the difference from controls was no longer statistically significant at 12 months. A weak correlation existed between FPI and pain (*p* < 0.05); no correlation was found with functional or alignment outcomes. Foot posture provides limited longitudinal insight; broader biomechanical assessment is advised.

## Introduction

 Knee osteoarthritis (OA) is a progressive musculoskeletal condition characterized by the degeneration of joint structures, including articular cartilage, subchondral bone, menisci, and surrounding soft tissues. These pathological changes often manifest clinically as joint pain, swelling, stiffness, and reduced range of motion, significantly affecting functional mobility and quality of life^1–3^. In advanced stages of knee OA, patients frequently present with lower limb malalignment, muscle weakness, and joint instability, contributing to altered movement patterns and increased mechanical stress on surrounding joints^4,5^. One such area of compensatory adaptation is the foot, which plays a critical role in maintaining posture, balance, and dynamic alignment of the lower extremity^6–8^.

The foot acts as the terminal base of support for the lower limb kinetic chain. Its posture and alignment can significantly influence, and be influenced by, proximal joint mechanics, particularly in the presence of deformities or pathologies such as knee OA. Alterations in foot posture including increased pronation, supination, or midfoot collapse, are often observed in individuals with varus or valgus knee deformities, as the foot adapts to maintain overall balance and stability. Such compensation, although functional in the short term, may lead to long-term biomechanical inefficiencies, increased energy expenditure during ambulation, and the development of secondary joint problems in the ankle, hip, or spine^9–11^.

Total knee arthroplasty (TKA) is widely considered the gold standard surgical intervention for end-stage knee OA when conservative measures fail to provide relief. TKA aims to correct joint deformity, relieve pain, and restore function^12,13^. However, while much focus has been placed on changes in limb alignment around the knee post-surgery, less attention has been given to the biomechanical outcomes in distal joints such as the foot. Despite successful surgical correction of knee alignment, compensatory foot postural changes that develop preoperatively may persist or undergo further adaptation, potentially impacting overall rehabilitation outcomes and long-term joint health^7,10^.

Foot posture serves as a clinically accessible and biomechanically relevant indicator of lower limb alignment and weight-bearing distribution. The use of standardized assessment tools, such as the Foot Posture Index (FPI), allows for objective evaluation of foot structure in multiple planes^14^. Given the kinetic chain relationship between the hip, knee, and foot, postural adaptations in the foot could reflect changes in limb alignment following TKA, thus providing insight into the structural integrity and functional restoration achieved through surgical and rehabilitative interventions. Moreover, abnormal foot posture may be associated with altered gait mechanics, increased joint loading, and persistent discomfort even after TKA, highlighting the need for its inclusion as a postoperative assessment parameter^6,9,10^.

To date, only a limited number of studies have evaluated foot posture as a biomechanical outcome following TKA, and these have typically focused on short-term changes or have not examined how foot posture relates to patient-reported outcomes such as pain or function. Existing evidence suggests that compensatory pronation or supination may persist even after surgical correction of knee alignment, but the long-term trajectory of these adaptations remains unclear. By examining foot posture over a year and correlating it with pain and lower-extremity function, the present study seeks to address these gaps and provide a more comprehensive understanding of distal biomechanical responses to TKA. We hypothesized that individuals with severe knee OA would exhibit altered foot posture preoperatively compared to healthy controls, and that foot posture would change following TKA in response to changes in lower limb alignment. Hence, the objective of this study was to assess foot posture as an outcome measure of biomechanical adaptation before and after TKA and to examine its correlation with pain and lower extremity function in individuals with severe knee OA.

## Materials and methods

### Study design and participants

This is a prospective, longitudinal single-centre study performed at the Department of Physiotherapy and Department of Orthopaedics, Kasturba Hospital, Manipal, Karnataka, India. Ethical approval was sought by the Institutional Ethics Committee (IEC) of Kasturba Hospital, Manipal (IEC1-20-2022). The study protocol was registered in the Clinical Trial Registry – India (CTRI/2022/07/043642). The study was conducted in accordance with the World Medical Association Declaration of Helsinki – Code of Ethics. In addition, we confirm that all methods were performed in accordance with the relevant guidelines and regulations. The Strengthening the Reporting of Observational Studies in Epidemiology (STROBE) guidelines were followed^15^.

Seventy-seven participants with severe knee OA who underwent TKA, and seventy-seven healthy controls were recruited to compare the TKA data. The sample size was calculated using the formula n = (Z₁₋⍺/2)² × σ² / d², assuming Z = 1.96 (95% CI), SD = 16, and minimal clinically relevant difference = 4. This yielded a required sample of 61, increased to 77 accounting for 20% attrition. The sample size for age- and sex-matched control group was *n* = 77^16^.

The inclusion criterion for the TKA group was patients aged 50–80 years of either sex with primary severe (Kellgren–Lawrence grade 4) knee OA who were scheduled for TKA using the medial parapatellar approach. The inclusion criteria for the control group were healthy individuals who did not have any current lower extremity orthopedic injuries or neurological, immunological, inflammatory, or cardiovascular diseases and were aged 50–80 years of either sex. Individuals who had inflammatory arthritis with multiple joint involvement, neurological deficits, or posttraumatic arthritis and those who declined to participate were excluded from the study^16^.

### Instrumentation and procedure

The screening of the participants was performed as per the eligibility criteria. The demographic information of the participants, including age (in years), sex, height (in cm), weight (in kg), and body mass index (BMI) (in kg/m2), was collected. The participants were informed about the procedure of the study, and their consent was acquired. The outcomes were assessed by a musculoskeletal physical therapist with clinical experience in knee assessment and rehabilitation. Foot posture, pain, functional outcomes and quadriceps (q) angle were assessed using relevant clinical tools. Measurements were taken preoperatively (one day before surgery) and at the 6th week, 3rd month, 6th month and 12th month following TKA^16^.

### Intervention program

All the participants underwent a standard postoperative TKA rehabilitation program post-TKA^17,18^. The standard rehabilitation programme consisted of strengthening exercises, mobility-based and stretching exercises, functional training, balance training, and aerobic conditioning. The stage 1 exercises were administered from day 1 to 2 weeks in the early function phase following TKA. Stage 2 exercises were administered in the progressive function phase from 3 to 6 weeks following TKA. Stage 3 exercises were administered in the advanced function phase from 7 to 12 weeks following TKA. The rehabilitation exercises progressed according to the postoperative stages^17^.

### Foot posture assessment before and after TKA

**Fig. 1:**
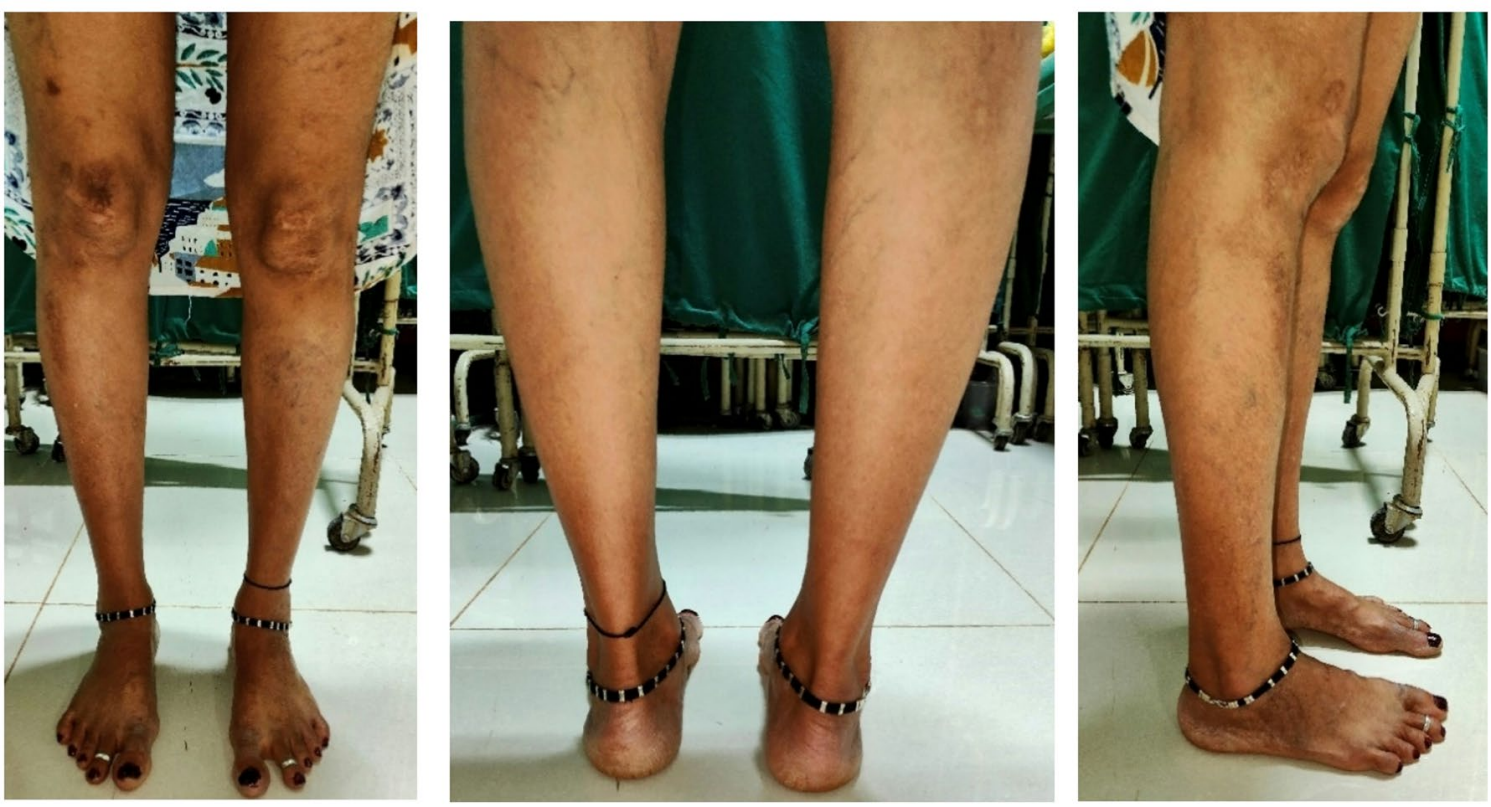
FPI assessment position for the anterior view, posterior view and lateral views.

**Fig. 2:**
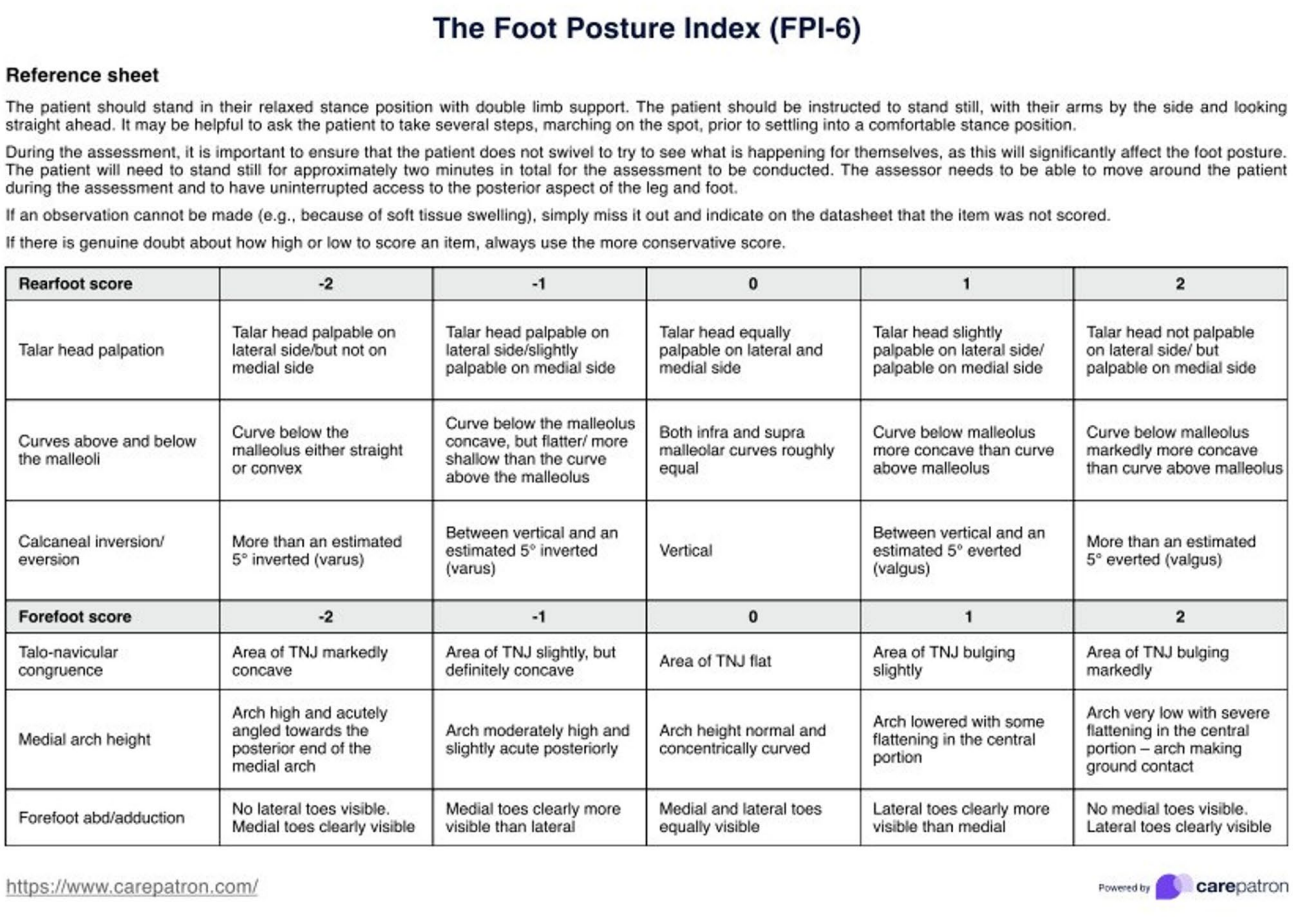
Foot posture index (FPI-6).

The FPI is a clinical tool used to assess the overall posture of the foot in the standing position. The assessment involved observing and scoring six criteria: talar head palpation, curves above and below the lateral malleoli, inversion/eversion of the calcaneus, bulging in the region of the talonavicular joint, congruence of the medial longitudinal arch, and abduction/adduction of the forefoot on the rearfoot. Each criterion is scored on a scale from − 2 (indicative of a supinated foot) to + 2 (indicative of a pronated foot), with a total score ranging from − 12 to + 12. The patient stood relaxed in a natural posture while the therapist evaluated each component (Fig. 1). The cumulative FPI score classifies the foot as highly supinated (–12 to − 5), supinated (–4 to − 1), neutral (0 to + 5), pronated (+ 6 to + 9), or highly pronated (+ 10 to + 12), aiding in diagnosis and treatment planning^14,19^. FPI is a reliable and valid clinical tool for assessing multi-planar foot posture, with demonstrated good inter-rater and intra-rater reliability^20–24^ (Fig. 2).

### Pain and lower extremity function assessment before and after TKA

Pain severity was evaluated using the Numerical Pain Rating Scale (NPRS), which ranges from 0 to 10, with scores ranging from 0 (no pain) to 10 (worst pain possible)^25,26^. This scale has been shown to be reliable in OA, and is recommended for use in knee OA clinical trials^27,28^. The Lower Extremity Functional Scale (LEFS) was used to assess patients’ initial function, track their progress, and evaluate outcomes in individuals with lower extremity impairments. It has excellent internal reliability (α = 0.96)^29^. The timed-up and go (TUG) test was used to measure the time required for an individual to rise from an armchair (seat height: 46 cm), walk 3 m, turn, and return to the chair without physical assistance. This test exhibited excellent inter-rater and intra-rater reliability, with an intraclass correlation coefficient (ICC) of 0.99^30^. The 6-minute walk test (6MWT) assesses the total distance walked in meters over six minutes. Following knee arthroplasty, it has been validated as a measure of functional performance and endurance, demonstrating excellent test‒retest reliability (ICCs: 0.95–0.97) and a low coefficient of variation (10.4%)^31,32^.

### Quadriceps angle (Q-angle) before and after TKA

The participants laid supine on a couch with a straight spine and legs parallel to each other with the quadriceps relaxed and the knees fully extended. The hip joints and feet were in a neutral position. The pelvis was squared, both the anterior superior iliac spines (ASISs) were at the same level, and the lower limbs were in a plane at 90 ◦ to the line joining the two ASISs. The ASIS, tibial tubercle, and center of the patella bony landmarks were adequately exposed while the measurements were taken and marked. Palpation and visual estimation were used to locate the anatomical landmarks for measurement. Two lines were drawn, one from the ASIS to the center of the patella and the other from the center of the patella to the center of the tibial tubercle. The acute angle formed between the two lines was measured using a goniometer^33^.

### Statistical analysis

The data were analyzed using Jamovi 2.6.23. Demographic characteristics were summarized using descriptive statistics. The values of the FPIs obtained are shown as the means and standard deviations (SDs). Friedman test was performed to compare the FPI scores before TKA, and at the 6th week, 3rd month, 6th month and 12th month after TKA. The correlations of the FPI score with pain, functional outcomes and q-angle were determined using Pearsons’s correlation coefficient. The level of statistical significance was set as *p* ≤ 0.05.

## Results

### Demographics

Seventy-seven individuals (47 females and 30 males) with severe knee OA, with a mean age of 66.93 (SD = 7.1) years and 77 healthy controls (45 females and 32 males), with a mean age of 62.78 (SD = 9.64) years, were included in the study. The mean duration of knee pain pre-TKA was 15.9 (SD = 4.3) months. The demographic details are shown in Table 1.

**Table 1:** Demographic & anthropometric characteristics of TKA group and control group participants.

Variables		TKA group (*n* = 77)	Control group (*n* = 77)
Age (in years) (Mean ± SD)		66.93 ± 7.1	62.78 ± 9.64
Gender (Female, Male)	Females (n, %)	47 (61.03%)	45 (58.44%)
	Males (n, %)	30 (38.96%)	32 (41.55%)
Height (in cm) (Mean ± SD)		155.8 ± 8.45	157.43 ± 6.12
Weight (in kg) (Mean ± SD)		69.21 ± 14.1	65.8 ± 12.32
BMI (in kg/m2) (Mean ± SD)		28.36 ± 11.3	25.78 ± 10.21
Leg dominance (Right, Left)	Right (n, %)	72 (93.5%)	69 (89.64%)
	Left (n, %)	5 (6.49%)	8 (10.38%)

*TKA: Total Knee Arthroplasty; cm: centimeter; kg: kilogram; BMI: Body Mass Index; SD: Standard Deviation.

### Comparison of FPI scores between individuals with knee OA and healthy controls

**Fig. 3:**
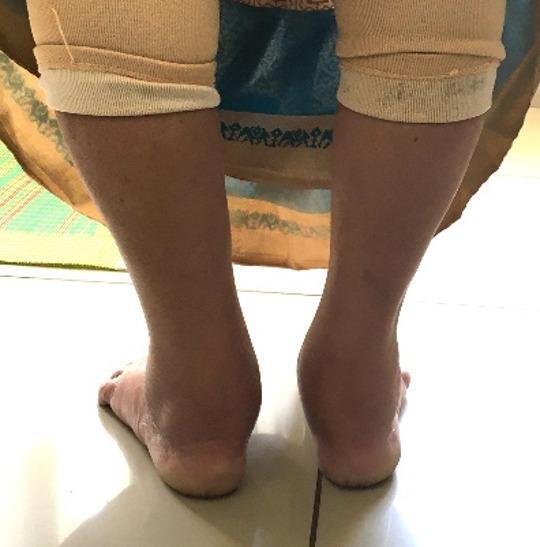
Foot posture assessment in individuals with knee OA.

The mean FPI score was 1.52 (SD = 2.5) in individuals with knee OA before TKA and 0.70 (SD = 2.2) in healthy controls. A significant (*p* = 0.03) difference was noted between the FPI scores of individuals with knee OA and healthy controls. Compared with controls, individuals with knee OA demonstrated mildly pronated foot in the FPI. (Table 2) (Fig. 3).

**Table 2:** FPI scores for pre-TKA and control groups.

Outcome measure	Pre-TKA (*n* = 77)	Controls (*n*= 77)	MD	95% CI	% difference	*p*-value
FPI	1.52 ± 2.5	0.70 ± 2.2	0.82	[0.08, 1.56]	53.95%	0.03*

*Significant at *p* ≤0.05; SD: standard deviation; TKA: total knee arthroplasty; MD: Mean difference; SEM: standard error of the difference; 95% CI: 95% confidence interval of the difference; FPI: foot posture index.

### Comparison of FPI scores in individuals with knee OA before and after TKA

The mean FPI score was 1.52 (SD = 2.5) in individuals with knee OA before TKA. Post-TKA, the mean FPI score was 1.56 (SD = 2.9) at the 6th week, 1.58 (SD = 2.7) at the 3rd month, 1.54 (SD = 2.15) at the 6th month and 1.5 (SD = 2.26) at the 12th month. No significant difference was noted between the FPI score in individuals with knee OA before TKA and at follow-up timepoints after TKA (Table 3).

**Table 3:** FPI scores for pre-TKA and post-TKA.

Outcome measure	Pre-TKA (*n* = 77)	Post-TKA – 6th w (*n* = 77)	Post-TKA – 3rd m (*n* = 77)	Post-TKA – 6th m (*n* = 56)	Post-TKA – 12th m (*n* = 28)	*p*-value
FPI	1.52 ± 2.5	1.56 ± 2.9	1.58 ± 2.71	1.54 ± 2.15	1.5 ± 2.26	0.06

*Significant at *p* ≤ 0.05; SD: standard deviation; TKA: total knee arthroplasty; w: week; m: month; FPI: foot posture index.

### Comparison of FPI scores between individuals with knee OA after TKA and healthy controls

The mean FPI score was 1.54 (SD = 2.15) in individuals with knee OA 6 months after TKA, 1.5 (SD = 2.26) 12 months after TKA and 0.70 (SD = 2.2) in healthy controls. A significant difference (*p* = 0.03) was noted between the FPI score at the 6th month after TKA in individuals with knee OA and healthy controls. However, there was no significant difference noted between the FPI score at the 12th month after TKA and healthy controls (Table 4).

**Table 4:** FPI scores for post-TKA and control groups.

Outcome measure	Controls (*n* = 77)	Post-TKA	MD	95% CI	% change	*p* - value
FPI	0.70 ± 2.2	1.54 ± 2.15 (6th m, *n* = 54)	−0.84	[− 1.60, − 0.08]	54.55% (At 6th m)	0.03*
	0.70 ± 2.2	1.5 ± 2.26 (12th m, *n* = 28)	−0.80	[− 1.80, 0.20]	53.33% (At 12th m)	0.12

*Significant at *p* ≤ 0.05; SD: standard deviation; TKA: total knee arthroplasty; w: week; m: month; FPI: foot posture index.

### Correlation of the FPI score with pain and function in individuals with knee OA following TKA

Pain levels demonstrated a consistent and significant reduction following TKA (7.97 ± 2.48 to 2.36 ± 1.45, *p* < 0.001) (Table 5). Compared to the preoperative mean NPRS score of 7.97 (SD = 2.48), pain decreased by approximately 46.3% at the 5th postoperative day, 52.8% at the 6th week, 57.8% at the 3rd month, 64.0% at the 6th month, and 70.4% at the 12th month, indicating progressive and sustained pain relief over time (*p* < 0.001). Functional performance, as assessed by the LEFS, showed notable improvement post-TKA (24.75 ± 6.28 to 60.25 ± 5.1, *p* < 0.001) (Table 5). The mean LEFS score increased from a baseline of 24.75 to 32.2 at the 6th week (a 30.1% increase), 46.8 at the 3rd month (an 89.1% increase), 58.5 at the 6th month (a 136.4% increase), and 60.25 at the 12th month (a 143.4% increase), reflecting a substantial and sustained functional recovery (*p* < 0.001).

**Table 5:** Mean (± SD) pain and lower extremity functional outcome scores for pre-TKA and post-TKA.

Outcome measure	Pre-TKA (*n* = 77)	Post-TKA – 6th w	Post-TKA – 3rd m	Post-TKA – 6th m	Post-TKA – 12th m	F	*p*-value
NPRS	7.97 ± 2.48	3.76 ± 1.4	3.36 ± 1.40	2.87 ± 1.36	2.36 ± 1.45	170.14	< 0.001*
LEFS	24.75 ± 6.28	32.2 ± 7.1	46.8 ± 6.9	58.5 ± 5.8	60.25 ± 5.1	226.65	< 0.001*
TUG – time (s)	26.23 ± 8.81	25.5 ± 11.1	20.5 ± 7.1	16.3 ± 4.7	14.3 ± 3.7	15.38	< 0.001*
6MWT – distance (m)	159 ± 38.84	172 ± 49.1	306 ± 36.6	452 ± 76.6	475.3 ± 49.7	9.87	0.001*
Q-angle (in degrees)	19.56 ± 2.01	16.45 ± 1.96	13.91 ± 1.93	13.36 ± 1.82	13.33 ± 1.97	114.96	< 0.001*

* Significant at *p* ≤ 0.05; SD: standard deviation; TKA: total knee arthroplasty; NPRS: numerical pain rating scale; LEFS: lower extremity functional scale; TUG: timed up and go; 6MWT: 6-minute walk test; Q-angle: quadriceps angle; F: Fisher’s F statistic; s: seconds; m: meters; w: week; m: month.

The mean TUG time improved from a baseline of 26.23 s to 25.5 s at the 6th week (a 2.86% improvement), 20.5 s at the 3rd month (a 27.9% improvement), 16.3 s at the 6th month (a 60.92% improvement), and 14.3 s at the 12th month (an 83.36% improvement), demonstrating a progressive and clinically meaningful enhancement in mobility and functional speed (*p* < 0.001). The mean 6MWT distance increased from 159 m at baseline to 172 m at the 6th week (a 7.56% increase), 306 m at the 3rd month (a 48.04% increase), 452 m at the 6th month (a 64.73% increase), and 475.3 m at the 12th month (a 66.54% increase), indicating a substantial and sustained improvement in walking endurance over time (*p* = 0.001).

A weak linear correlation (*r* = 0.2; *p* < 0.05) was noted between the FPI score and pain before TKA and at 12th month post-TKA suggesting a small effect size. No significant correlation (*p* > 0.05) was found between the FPI and LEFS, TUG and 6MWT scores pre-TKA and at the 12th month post-TKA.

### Correlation of the FPI score with Q-angle in individuals with knee OA following TKA

Our study demonstrated a significant reduction in the Q-angle across all postoperative timepoints (*p* < 0.001). The mean Q-angle decreased from 19.56° ± 2.01 pre-TKA to 16.45° ± 1.96 at 6 weeks, 13.91° ± 1.93 at 3 months, 13.36° ± 1.82 at 6 months, and 13.33° ± 1.97 at 12 months post-TKA. Correspondingly, the percentage reductions in Q-angle were 28.67% at 3 months, 31.49% at 6 months, and 31.64% at 12 months, indicating a sustained and stable improvement over time. There was a weak linear correlation between the FPI score and the Q-angle before TKA (*r* = 0.2; *p* < 0.05). However, no significant correlation was observed between these variables at any postoperative timepoint following TKA, indicating that improvements in proximal knee alignment did not translate into changes in distal foot posture.

## Discussion

The objective of this study was to evaluate foot posture as an outcome measure before and after TKA in individuals with severe knee OA and to determine the correlation of the FPI score with pain and lower extremity function following TKA.

### Clinical measurement of foot posture before and after TKA

The significant difference in FPI scores between individuals with knee OA and healthy controls suggests that chronic knee joint degeneration may influence foot posture. Specifically, the higher FPI scores in individuals with OA than in healthy individuals indicate a tendency toward a mildly pronated foot posture, which is in line with previous reports^14,34,35^ and reflects compensatory biomechanical strategies developed over time to reduce pain and to maintain mobility.

Interestingly, despite undergoing TKA, there was no statistically significant change in FPI scores at any postoperative time point up to 12 months, indicating that surgical correction of the knee alone may not be sufficient to influence established foot posture. This finding may reflect the persistence of neuromuscular adaptations and learned movement patterns developed during the chronic phase of OA, which are not automatically reversed with surgical intervention^7,36,37^. However, the reduced number of participants at later follow-ups may have limited the ability to detect subtle longitudinal changes. Given the considerable attrition at the 6-month and 12-month follow-ups, the reduced sample size poses a risk of attrition bias, which may have influenced the stability and generalizability of the longitudinal trends observed. To address this, baseline characteristics were compared between participants who completed follow-up assessments and those lost to follow-up, which showed no major systematic differences; however, the possibility of unmeasured bias cannot be fully excluded. Some outcome measures such as NPRS could be collected via telephone, resulting in full follow-up data, whereas foot posture assessments required in-person visits, contributing to the differential completion rates.

Several factors could explain why foot posture remains altered following TKA. First, while TKA addresses structural and functional issues at the knee joint, it does not directly target the foot or ankle complex. Chronic adaptations in the foot, such as ligamentous laxity or altered muscle activation, may persist unless they are specifically addressed through rehabilitation^7,38^. Furthermore, the duration of preoperative symptoms, which averaged nearly 16 months, may contribute to the entrenchment of compensatory mechanisms that are slow to change, even with improved knee function and reduced pain. Even 12 months after TKA, FPI scores differ significantly between individuals with knee OA and healthy controls, suggesting that foot posture may remain unaffected by surgical correction of the knee joint. This highlights the need to recognize and manage secondary musculoskeletal adaptations, such as altered foot posture, that may persist despite successful knee realignment^39–41^.

### Correlation of FPI scores with pain and function following TKA

Although pain significantly improved after TKA, the weak statistically significant but clinically negligible correlation between FPI scores and pain suggests that pain relief alone does not directly translate to normalization of foot posture. Additionally, no significant correlation was found between FPI and functional outcomes, indicating that improvements in functional ability post-TKA may occur independently of changes in foot posture. This may be attributed to the fact that functional recovery is influenced by multiple factors, including muscle strength, joint stability, proprioception, and patient engagement in rehabilitation, rather than static foot alignment alone. These findings highlight the importance of addressing foot posture and gait mechanics through comprehensive rehabilitation programs that go beyond knee joint recovery, particularly in patients exhibiting persistent postural deviations despite surgical intervention^38,42,43^.

These findings suggest that knee OA is associated with a mild shift toward pronated foot posture, which does not significantly change following TKA, even with substantial pain reduction. This could be due to long-standing neuromuscular and biomechanical adaptations that persist unless specifically targeted. Strategies can be developed to improve foot posture, restore lower limb biomechanics, and enhance long-term function.

### Correlation of FPI scores with Q-angle following TKA

The comparison of mean trends between the FPI score and the Q-angle suggests an important but complex relationship between proximal knee alignment and distal foot posture in individuals with severe knee OA. Preoperatively, participants demonstrated both a higher Q-angle and a more pronated foot posture, indicating that altered knee alignment may contribute to compensatory foot mechanics. This pattern is consistent with the biomechanical understanding that genu varum and increases in Q-angle can lead to altered loading patterns along the lower limb kinetic chain, often resulting in pronation as a compensatory strategy to maintain balance and distribute forces during weight-bearing. Although a weak linear correlation was observed between the FPI score and Q-angle before TKA, this relationship appears to reflect long-standing adaptations developed in response to chronic knee malalignment.

Following TKA, however, no meaningful correlation was noted between Q-angle and foot posture. Despite a substantial reduction in Q-angle, reflecting improved proximal realignment—the FPI scores remained largely unchanged throughout postoperative follow-up. This dissociation indicates that correction of knee alignment alone does not automatically restore distal alignment or reverse foot posture compensations that have developed over years of degenerative joint changes. Persistent foot pronation despite improvements in Q-angle may arise from structural adaptations, ligamentous laxity, altered neuromuscular control, or habitual gait strategies that are not directly modified by TKA. These findings emphasize the need for targeted rehabilitation strategies addressing both knee and foot biomechanics, as distal compensations may remain entrenched even after surgical correction of proximal alignment. Integrating foot and ankle assessment into post-TKA rehabilitation may enhance lower limb biomechanical efficiency and long-term functional outcomes.

### Clinical implications

This study offers important clinical insights into the persistence of altered foot posture following TKA in individuals with knee OA. The findings highlight the value of using accessible and clinically feasible tools such as the FPI to monitor lower limb alignment beyond the knee joint. Although TKA effectively improves joint structure, relieves pain, and enhances function, our results suggest that associated compensatory changes in foot posture particularly a tendency toward pronation may not resolve postoperatively without specific intervention.

These persistent postural alterations may contribute to inefficient gait mechanics or uneven load distribution, even after successful surgical correction. This underscores the importance of a holistic, kinetic-chain-based approach in post-TKA rehabilitation, one that considers the interdependent nature of the lower extremity kinetic chain. Clinical evaluation should be routinely extended to the foot and ankle, not only for diagnostic purposes but also to inform targeted rehabilitation planning. Rehabilitation programs following TKA should incorporate strategies aimed at improving foot and ankle function, including strengthening exercises, neuromuscular control training, and gait retraining. In cases of persistent foot misalignment, the use of orthotics or supportive footwear may also be beneficial. Such comprehensive interventions can promote better biomechanical efficiency and potentially reduce the risk of secondary complications or future joint strain.

We have also added explicit commentary on the limited clinical significance of the statistically significant differences and clarified that the persistence of a neutral or near-neutral foot posture post-TKA may reflect a stable and functionally optimal alignment that does not necessarily change following surgery. Moreover, the lack of a strong correlation between foot posture and improvements in pain or function highlights that traditional outcome measures may not fully capture the scope of biomechanical adaptation. Therefore, integrating FPI assessment with routine follow-up could serve as an additional metric for identifying individuals who may benefit from extended or specialized rehabilitation. Overall, these findings support a more integrated and personalized approach to post-TKA care, one that addresses not only pain and mobility at the knee, but also the functional alignment of the entire lower limb to optimize long-term outcomes and patient quality of life.

### Limitations

The reduction in sample size at the six-month (*n* = 54) and twelve-month (*n* = 28) follow-ups due to participant attrition may have limited the statistical power of the study and reduced the generalizability of the longitudinal findings. Some outcome measures, such as the NPRS, could be collected via telephone and therefore had complete follow-up data. However, assessments that required in-person evaluation, such as the FPI, resulted in smaller sample sizes at later time points. Although the FPI is a reliable and clinically feasible tool, it remains semiquantitative and susceptible to interrater variability, which may introduce measurement bias.

A key limitation is that the study did not include comprehensive knee alignment measures, for example the hip knee ankle angle or even basic alignment categories such as varus, valgus, or neutral. The absence of these measures limits the ability to fully examine how proximal knee alignment relates to distal foot posture after TKA and restricts the depth of biomechanical interpretation. The study also did not incorporate dynamic biomechanical assessments, which prevented evaluation of the relationship between foot posture and dynamic knee loading. Finally, potential confounding factors such as differences in rehabilitation protocols, footwear habits, and physical activity levels were not controlled for, and any of these may influence foot posture independently of surgical correction.

### Future recommendations

Future studies may explore the relationship between foot posture and lower limb alignment parameters such as the hip-knee-ankle (HKA) angle or tibial torsion to provide a more comprehensive understanding of compensatory biomechanical adaptations post-TKA. Additionally, long-term follow-up beyond 12 months could offer valuable insights into whether persistent changes in foot posture stabilize, worsen, or improve over time. Future rehabilitation protocols following TKA should consider routine foot posture assessments and address compensatory postural changes. Further research incorporating objective measures such as dynamic knee loading, knee adduction moment, gait analysis, plantar pressure distribution, or dynamic balance testing may help correlate foot posture alterations with functional gait outcomes.

Future research should also incorporate baseline and postoperative knee alignment information, including simple clinical classifications such as varus, neutral, or valgus alignment, to allow clearer interpretation of how proximal alignment influences long-term foot posture and functional recovery following TKA. Instrumented 3D gait analysis to link foot posture to knee joint mechanics. Dynamic plantar pressure measurements to assess foot function during walking. A more focused research question that tests a specific hypothesis about how dynamic foot function influences TKA recovery. Studies evaluating the effectiveness of specific post-TKA rehabilitation strategies, including foot orthoses, gait retraining, and ankle-foot strengthening, could also help inform evidence-based clinical guidelines for enhancing recovery, optimizing lower limb biomechanics and improving long-term functional outcomes.

## Conclusions

This study evaluated foot posture before and after TKA and examined its relationship with pain and functional recovery in individuals with severe knee OA. Although individuals with knee OA demonstrated slightly higher FPI scores than healthy controls preoperatively, these values remained within the neutral range and showed no meaningful change at any postoperative follow-up. The small, statistically significant differences observed had limited clinical relevance, indicating that foot posture deviations are minimal and largely stable over time. Furthermore, only a weak and clinically negligible association was found between FPI and postoperative pain, and no relationship was observed with functional outcomes or improvements in knee alignment.

These findings suggest that static foot posture, as measured by the FPI, does not reflect the substantial gains in pain relief, mobility, and function typically achieved after TKA and is not responsive to surgical correction of knee alignment. While foot posture assessment remains valuable for comprehensive lower-limb evaluation, it appears to be neither sensitive nor informative as a longitudinal outcome measure following TKA. Future work integrating dynamic biomechanical assessments may better elucidate distal compensations and guide more holistic rehabilitation strategies.

## Data Availability

The datasets generated during and/or analysed during the current study are available from the corresponding author on reasonable request.

## Abbreviations

OA: Osteoarthritis
TKA: Total knee arthroplasty
FPI: Foot posture index
IEC: Institutional Ethics Committee
CTRI: Clinical Trial Registry – India
STROBE: Strengthening the reporting of observational studies in epidemiology
cm: Centimetre
kg: Kilogram
kg/m^2^: Kilogram per meter square
BMI: Body mass index
NPRS: Numerical pain rating scale
LEFS: Lower extremity functional scale
TUG: Timed-up and go
6MWT: Six-minute walk test
Q-angle: Quadriceps angle
ICC: Intraclass correlation coefficient
ASIS: Anterior superior iliac spines
SD: Standard deviation

## References

[1] Krasnokutsky, S., Attur, M., Palmer, G., Samuels, J. & Abramson, S. B. Current concepts in the pathogenesis of osteoarthritis. *Osteoarthr. Cartil.* **16**, S1–S3. 10.1016/j.joca.2008.06.025 (2008). 10.1016/j.joca.2008.06.025 18723377

[2] Vitaloni, M. et al. Global management of patients with knee osteoarthritis begins with quality of life assessment: a systematic review. *BMC Musculoskelet. Disord*. **20** (1), 493. 10.1186/s12891-019-2895-3 (2019). 31656197 10.1186/s12891-019-2895-3 PMC6815415

[3] Nelson, A. E. Osteoarthritis year in review 2017: clinical. *Osteoarthr. Cartil.* **26** (3), 319–325. 10.1016/j.joca.2017.11.014 (2018). 10.1016/j.joca.2017.11.014 PMC5835411 29229563

[4] Lewek, M. D., Rudolph, K. S. & Snyder-Mackler, L. Quadriceps femoris muscle weakness and activation failure in patients with symptomatic knee osteoarthritis. *J. Orthop. Res.* **22** (1), 110–115. 10.1016/S0736-0266(03)00154-2 (2004). 14656668 10.1016/S0736-0266(03)00154-2 PMC3073134

[5] Rice, D. A., McNair, P. J. & Lewis, G. N. Mechanisms of quadriceps muscle weakness in knee joint osteoarthritis: the effects of prolonged vibration on torque and muscle activation in Osteoarthritic and healthy control subjects. *Arthritis Res. Ther.* **13** (5), R151. 10.1186/ar3467 (2011). 21933392 10.1186/ar3467 PMC3308081

[6] Chen, Z. et al. Association between foot posture asymmetry and static stability in patients with knee osteoarthritis: a case-control study. *Biomed. Res. Int.* **2020** (1). 10.1155/2020/1890917 (2020). 10.1155/2020/1890917 PMC7294354 32596282

[7] Farrokhi, S., Voycheck, C. A., Tashman, S. & Fitzgerald, G. K. A Biomechanical perspective on physical therapy management of knee osteoarthritis. *J. Orthop. Sports Phys. Therapy*. **43** (9), 600–619. 10.2519/jospt.2013.4121 (2013). 10.2519/jospt.2013.4121 23756435

[8] Wei, Z., Zeng, Z., Liu, M. & Wang, L. Effect of intrinsic foot muscles training on foot function and dynamic postural balance: A systematic review and meta-analysis. *PLoS One*. **17** (4), e0266525. 10.1371/journal.pone.0266525 (2022). 35442981 10.1371/journal.pone.0266525 PMC9020712

[9] Powers, C. M. The influence of abnormal hip mechanics on knee injury: A Biomechanical perspective. *J. Orthop. Sports Phys. Therapy*. **40** (2), 42–51. 10.2519/jospt.2010.3337 (2010). 10.2519/jospt.2010.3337 20118526

[10] Metsavaht, L. et al. A biokinetic approach in primary knee osteoarthritis prevention and management–exploring movement profiles and kinetic chain interactions: current concepts. *J. ISAKOS*. **10**, 100381. 10.1016/j.jisako.2024.100381 (2025). 39743209 10.1016/j.jisako.2024.100381

[11] Ohi, H. et al. Association of frontal plane knee alignment with foot posture in patients with medial knee osteoarthritis. *BMC Musculoskelet. Disord*. **18** (1), 246. 10.1186/s12891-017-1588-z (2017). 28592232 10.1186/s12891-017-1588-z PMC5463360

[12] Aslam, M. et al. Approach to total knee replacement: A randomized double blind study between medial parapatellar and midvastus approach in the early postoperative period in Asian population. *J. Knee Surg.* **30** (08), 793–797. 10.1055/s-0036-1597978 (2017). 28086244 10.1055/s-0036-1597978

[13] Hall, M. C. Relative impact of radiographic osteoarthritis and pain on quadriceps strength, proprioception, static postural sway and lower limb function. *Ann. Rheum. Dis.* **65** (7), 865–870. 10.1136/ard.2005.043653 (2005). 16308342 10.1136/ard.2005.043653 PMC1798212

[14] N, F. E. A. A positive association between foot posture index and medial compartment knee osteoarthritis in Moroccan people. *Open. Rheumatol. J.* **8** (1), 96–99. 10.2174/1874312901408010096 (2014). 25553141 10.2174/1874312901408010096 PMC4279032

[15] von Elm, E. et al. The strengthening the reporting of observational studies in epidemiology (STROBE) statement: guidelines for reporting observational studies. *J. Clin. Epidemiol.* **61** (4), 344–349. 10.1016/j.jclinepi.2007.11.008 (2008). 18313558 10.1016/j.jclinepi.2007.11.008

[16] Shetty, S., Maiya, G. A., Rao, K. G. M., Vijayan, S. & George, B. M. Quadriceps angle as an outcome measure for structural integrity following total knee arthroplasty in individuals with severe knee osteoarthritis. *J. Orthop.* **61**, 37–42. 10.1016/j.jor.2024.09.010 (2025). 39386419 10.1016/j.jor.2024.09.010 PMC11459452

[17] Aseer, P. A. L. Content validation of total knee replacement rehabilitation protocol in Indian population. *J. Clin. Diagn. Res.*10.7860/JCDR/2017/27528.10137 (2017). 10.7860/JCDR/2017/27528.10137 PMC5535470 28764280

[18] Zeni, J. A. & Snyder-Mackler, L. Early postoperative measures predict 1- and 2-Year outcomes after unilateral total knee arthroplasty: importance of contralateral limb strength. *Phys. Ther.* **90** (1), 43–54. 10.2522/ptj.20090089 (2010). 19959653 10.2522/ptj.20090089 PMC2802824

[19] Redmond, A. C., Crosbie, J. & Ouvrier, R. A. Development and validation of a novel rating system for scoring standing foot posture: the foot posture index. *Clin. Biomech. Elsevier Ltd*. **21** (1), 89–98. 10.1016/j.clinbiomech.2005.08.002 (2006). 10.1016/j.clinbiomech.2005.08.002 16182419

[20] Aydin Yağcioğlu, G. & Karapınar, M. Translation, validity, and reliability of the foot posture index (FPI-6) – Turkish version. *Physiother Theory Pract.* **40** (8), 1830–1836. 10.1080/09593985.2023.2207109 (2024). 10.1080/09593985.2023.2207109 37158698

[21] Morrison, S. C. & Ferrari, J. Inter-rater reliability of the foot posture index (FPI‐6) in the assessment of the paediatric foot. *J. Foot Ankle Res.* **2** (1). 10.1186/1757-1146-2-26 (2009). 10.1186/1757-1146-2-26 PMC2770503 19845961

[22] Aquino, M. R. C., Avelar, B. S., Silva, P. L., Ocarino, J. M. & Resende, R. A. Reliability of foot posture index individual and total scores for adults and older adults. *Musculoskelet. Sci. Pract.* **36**, 92–95. 10.1016/j.msksp.2018.02.002 (2018). 29428292 10.1016/j.msksp.2018.02.002

[23] Kyung, M. G. et al. Reliability and radiographic correlation of the foot posture index-6: a multi-rater analysis in symptomatic and asymptomatic individuals. *Diagnostics (Basel)* **15** (10). 10.3390/diagnostics15101214 (2025). 10.3390/diagnostics15101214 PMC12109674 40428206

[24] Yang, J. et al. Reliability and validity of foot posture index (FPI-6) for evaluating foot posture in participants with low back pain. *Sci. Rep.* **12** (1), 21168. 10.1038/s41598-022-22220-1 (2022). 36477012 10.1038/s41598-022-22220-1 PMC9729570

[25] Nugent, S. M., Lovejoy, T. I., Shull, S., Dobscha, S. K. & Morasco, B. J. Associations of pain numeric rating scale scores collected during usual care with research administered patient reported pain outcomes. *Pain Med.* **22** (10), 2235–2241. 10.1093/pm/pnab110 (2021). 33749760 10.1093/pm/pnab110 PMC8677438

[26] Salaffi, F., Stancati, A., Silvestri, C. A., Ciapetti, A. & Grassi, W. Minimal clinically important changes in chronic musculoskeletal pain intensity measured on a numerical rating scale. *Eur. J. Pain*. **8** (4), 283–291. 10.1016/j.ejpain.2003.09.004 (2004). 15207508 10.1016/j.ejpain.2003.09.004

[27] Bellamy, N. Outcome measurement in osteoarthritis clinical trials. *J. Rheumatol. Suppl.* **43**, 49–51 (1995). 7752137

[28] Bellamy, N. Osteoarthritis clinical trials: candidate variables and clinimetric properties. *J. Rheumatol.* **24** (4), 768–778 (1997). 9101516

[29] Binkley, J. M., Stratford, P. W., Lott, S. A. & Riddle, D. L. The lower extremity functional scale (LEFS): scale development, measurement properties, and clinical application. North American orthopaedic rehabilitation research network. *Phys. Ther.* **79** (4), 371–383 (1999). 10201543

[30] Podsiadlo, D. & Richardson, S. The timed up & go: A test of basic functional mobility for frail elderly persons. *J. Am. Geriatr. Soc.* **39** (2), 142–148. 10.1111/j.1532-5415.1991.tb01616.x (1991). 1991946 10.1111/j.1532-5415.1991.tb01616.x

[31] Parent, E. & Moffet, H. Comparative responsiveness of locomotor tests and questionnaires used to follow early recovery after total knee arthroplasty. *Arch. Phys. Med. Rehabil*. **83** (1), 70–80. 10.1053/apmr.2002.27337 (2002). 11782835 10.1053/apmr.2002.27337

[32] Steffen, T. M., Hacker, T. A. & Mollinger, L. Age- and Gender-Related test performance in Community-Dwelling elderly people: Six-Minute walk test, Berg balance Scale, timed up & go test, and gait speeds. *Phys. Ther.* **82** (2), 128–137. 10.1093/ptj/82.2.128 (2002). 11856064 10.1093/ptj/82.2.128

[33] (Kar) Nandi, M. et al. The ‘quadriceps angle’: correlation between clinical and radiographic measurements from a study in North Bengal. *J. Anat. Soc. India*. **62** (1), 68–72. 10.1016/S0003-2778(13)80016-0 (2013).

[34] Gosavi, P. M., Kolke, S. S., Chitre, J., Shyam, A. & Sancheti, P. Foot posture assessment in people with primary medial compartment knee osteoarthritis. *Physiotherapy - J. Indian Association Physiotherapists*. **15** (1), 12–16. 10.4103/PJIAP.PJIAP_10_20 (2021).

[35] Reilly, K. et al. The role of foot and ankle assessment of patients with lower limb osteoarthritis. *Physiotherapy* **95** (3), 164–169. 10.1016/j.physio.2009.04.003 (2009). 19635335 10.1016/j.physio.2009.04.003

[36] Heijink, A. et al. Biomechanical considerations in the pathogenesis of osteoarthritis of the knee. *Knee Surg. Sports Traumatol. Arthrosc.* **20** (3), 423–435. 10.1007/s00167-011-1818-0 (2012). 22173730 10.1007/s00167-011-1818-0 PMC3282009

[37] Tayfur, B., Charuphongsa, C., Morrissey, D. & Miller, S. C. Neuromuscular joint function in knee osteoarthritis: A systematic review and meta-analysis. *Ann. Phys. Rehabil Med.* **66** (2), 101662. 10.1016/j.rehab.2022.101662 (2023). 35364316 10.1016/j.rehab.2022.101662

[38] Al-Jabri, T., Brivio, A., Maffulli, N. & Barrett, D. Management of instability after primary total knee arthroplasty: an evidence-based review. *J. Orthop. Surg. Res.* **16** (1), 729. 10.1186/s13018-021-02878-5 (2021). 34930375 10.1186/s13018-021-02878-5 PMC8686357

[39] Butler, J. J., Mercer, N. P., Hurley, E. T., Azam, M. T. & Kennedy, J. G. Alignment of the hindfoot following total knee arthroplasty: A systematic review. *World J. Orthop.* **12** (10), 791–801. 10.5312/wjo.v12.i10.791 (2021). 34754835 10.5312/wjo.v12.i10.791 PMC8554349

[40] Levinger, P. et al. Foot posture in people with medial compartment knee osteoarthritis. *J. Foot Ankle Res.* **3** (1). 10.1186/1757-1146-3-29 (2010). 10.1186/1757-1146-3-29 PMC3020154 21162748

[41] Reilly, K. A., Barker, K. L., Shamley, D. & Sandall, S. Influence of foot characteristics on the site of lower limb osteoarthritis. *Foot Ankle Int.* **27** (3), 206–211. 10.1177/107110070602700310 (2006). 16539904 10.1177/107110070602700310

[42] Konnyu, K. J. et al. Rehabilitation for total knee arthroplasty. *Am. J. Phys. Med. Rehabil*. **102** (1), 19–33. 10.1097/PHM.0000000000002008 (2023). 35302953 10.1097/PHM.0000000000002008 PMC9464796

[43] Capin, J. J., Bade, M. J., Jennings, J. M., Snyder-Mackler, L. & Stevens-Lapsley, J. E. Total knee arthroplasty assessments should include strength and Performance-Based functional tests to complement range-of-motion and patient-reported outcome measures. *Phys. Ther.* **102** (6). 10.1093/ptj/pzac033 (2022). 10.1093/ptj/pzac033 PMC9393064 35358318

